# Changes in Landscape Greenness and Climatic Factors over 25 Years (1989–2013) in the USA

**DOI:** 10.3390/rs9030295

**Published:** 2017

**Authors:** Maliha S. Nash, James Wickham, Jay Christensen, Timothy Wade

**Affiliations:** 1 U.S. Environmental Protection Agency, Office of Research and Development, National Exposure Research Laboratory, Las Vegas, NV 89119, USA; Christensen.Jay@epa.gov; 2 U.S. Environmental Protection Agency, Office of Research and Development, National Exposure Research Laboratory, Research Triangle Park, NC 27711, USA; Wickham.james@epa.gov; 3 Retired; timw11@yahoo.com

**Keywords:** long-term monitoring, NDVI change, USA, direct factors, climatic factors, autoregression model

## Abstract

Monitoring and quantifying changes in vegetation cover over large areas using remote sensing can be achieved using the Normalized Difference Vegetation Index (NDVI), an indicator of greenness. However, distinguishing gradual shifts in NDVI (e.g., climate related-changes) versus direct and rapid changes (e.g., fire, land development) is challenging as changes can be confounded by time-dependent patterns, and variation associated with climatic factors. In the present study, we leveraged a method that we previously developed for a pilot study to address these confounding factors by evaluating NDVI change using autoregression techniques that compare results from univariate (NDVI vs. time) and multivariate analyses (NDVI vs. time and climatic factors) for 7,660,636 1 km × 1 km pixels comprising the 48 contiguous states of the USA, over a 25-year period (1989–2013). NDVI changed significantly for 48% of the nation over the 25-year period in the univariate analyses where most significant trends (85%) indicated an increase in greenness over time. By including climatic factors in the multivariate analyses of NDVI over time, the detection of significant NDVI trends increased to 53% (an increase of 5%). Comparisons of univariate and multivariate analyses for each pixel showed that less than 4% of the pixels had a significant NDVI trend attributable to gradual climatic changes while the remainder of pixels with a significant NDVI trend indicated that changes were due to direct factors. While most NDVI changes were attributable to direct factors like wildfires, drought or flooding of agriculture, and tree mortality associated with insect infestation, these conditions may be indirectly influenced by changes in climatic factors.

## 1. Introduction

Remote sensing data have been used by numerous researchers to monitor and quantify changes in vegetation cover over large areas for long-term time frames [1–8]. Vegetation change maps can inform environmental decision-makers of widespread general trends and can identify specific areas where land conditions are degrading or improving. The Normalized Difference Vegetation Index (NDVI), derived from the Advanced Very High Resolution Radiometer (AVHRR) satellite data, is a widely used indicator to evaluate vegetative condition over time [9–11], and is often referred to as an index of greenness. Changes in vegetation can be detected and quantified using NDVI in combination with historical data and expert knowledge, and this approach has been applied in a number of areas with diverse land cover, including: Oregon, USA [5,12], Europe [13], Morocco [14], the African Sahel [15,16], and globally [17]. NDVI has been used to identify gradual changes over decades [18,19], as well as more direct and rapid changes such as those caused by fire, agriculture, land clearing, and habitat restoration [20–22].

Distinguishing gradual shifts in NDVI (e.g., climate-related changes) versus direct and rapid changes (e.g., fire, land development) is challenging as changes can be confounded by variation associated with climatic factors and by time-dependent patterns. Climatic factors such as precipitation and temperature often strongly influence vegetation physiology and phenology and hence greenness [23,24]. NDVI has been shown to be related to climatic factors, particularly precipitation (e.g., [25–29]). Climatic factors may show a general pattern of change over time [30], and thus may account for a trend in NDVI in some areas. Moreover, to detect change in vegetation cover, it is also important to account for time-dependent patterns in NDVI, which are typically pronounced [5,29,31]. There have been several recent studies of the relationship between AVHRR NDVI trends and some climatic factors ([32–50]; Table 1). A few patterns emerge from examination of these studies: (1) past studies rely on global datasets with coarse spatial resolution (8 km resolution) [51]; (2) the majority of studies focus on the relationship between AVHRR NDVI and precipitation, with some studies relating AVHRR NDVI and temperature, and very few including other climatic factors; and (3) autoregressive techniques that control for serial correlation in the time series data are generally not included. In a few studies, the Durbin–Watson (DW) statistic was evaluated to test for the presence of serial correlation (e.g., [52]) and the non-parametric Mann–Kendall test was used to test for the existence of a monotonic trend, but controlling for the effects of serial correlation in detection of trends between AVHRR NDVI and time or climatic factors has not been a common practice.

This study addresses the gaps in past NDVI analyses, investigating long-term changes in AVHRR NDVI across the continental Unites States (CONUS) at a 1-km resolution, including multiple climatic factors in the analysis and accounting for serial autocorrelation. Unlike previous studies, the use of this statistical approach improves our ability to identify significant changes of NDVI (greenness) with time. Even more importantly, we identify trends in NDVI combining novel univariate (NDVI = time) and multivariate (NDVI = time + climatic factors) autoregressive models. These combined models allow us to differentiate between NDVI changes related to specific climatic factors and non-climatic factors across the CONUS. Comparison of the behaviors (i.e., significance) of individual pixels in the two models can be used to identify whether trends can be attributable to direct (land cover change, pest infestation, fire) or indirect factors. This work builds on a previous study in which these methods (per-pixel comparison of univariate and multivariate autoregressive model results) were tested using AVHRR NDVI time series data for the state of New Mexico, USA [8]. The objective of the current research is to extend the analyses in [8] to the entire continental U.S. Extension to the entire continental United States is worthwhile because changes related to both direct and indirect factors are not spatially stationary [53–55], and it is therefore unlikely that the spatial pattern of NDVI trends can be extrapolated from a small region [8] to the entirety of the continental United States. Additionally, our approach was developed with the intent of being more broadly applicable, regardless of land cover type.

## 2. Materials and Methods

### 2.1. Data

We used the 1 km × 1 km AVHRR NDVI produced by the U.S. Geological Survey Earth Resource Observation and Science (EROS) Center as our 25-year NDVI time series (1989–2013). The AVHRR 1 km × 1 km local area coverage (LAC) dataset is fully described by [56]. Similar to the commonly used global datasets (see Table 2 in [51]), pre-processing of the 1 km × 1 km AVHRR NDVI data include radiometric calibration and atmospheric correction to reduce or remove effects related to ozone, water vapor absorption and Rayleigh scattering. These data are based on the native, at-nadir 1 km × 1 km spatial resolution of the AVHRR sensor, rather than the on-board resampled 4 km × 4 km spatial resolution data on which the global AVHRR NDVI datasets are based, which likely reduces cloud contamination relative to the 4 km × 4 km data [56]. The 1 km × 1 km AVHRR NDVI data are biweekly maximum values composites [57] obtained from the daily overpasses, and the Clouds from AVHRR-Phase 1 (CLAVR-1) algorithm [58] is used to further reduce the effects of cloud contamination. Image-to-image registration is used to preserve the geometric integrity of the time series. Our dataset included 300 (12 months × 25 years; from January 1989–December 2013) NDVI observations per pixel. Description of the AVHRR LAC data is provided in the Supplementary Materials.

The main climatic parameters for the continental U.S. analyses are derived from the 4 km × 4 km Parameter-elevation Regressions on Independent Slopes Model [59] which incorporate field-based climate data with regressed interpolations. The 4 km × 4 km PRISM pixels were gridded into 1 km × 1 km using inverse distance weighting to match the spatial resolution of the AVHRR data (see [60]). The climatic factors provided by the PRISM data included monthly averages of minimum and maximum temperature, precipitation, and dew point temperature (more description of the Climatic factors data is provided in the Supplementary Materials). Previous research has shown that greenness is strongly associated with temperature and precipitation [10,11]. In addition, we derived an additional climatic parameter from the PRISM data, which was a one-month lag in precipitation data.

### 2.2. Statistical Methods

We conducted two types of analyses for each of the 7,660,636 1 km × 1 km pixels in the continental U.S. First, we conducted univariate autoregression of NDVI versus time to quantify the temporal trend (slope) for NDVI, and for each of the climatic parameters (e.g., precipitation versus time). The trend direction for each significant pixel was then mapped to identify geographic patterns of significance and trend direction. Second, we conducted a multivariate autoregression of NDVI versus time and climatic parameters to reveal the combined effects and relative contributions of climatic factors to significant NDVI trends. Time series regression (autoregression) was used in both analyses because errors in temporal data may be serially dependent, and if dependency exists, the standard error of the estimate (e.g., slope) would be inflated. Our autoregressive error model included a backstep function with up to 30 lags to account for serial dependence in errors. Autocorrelation function (ACF) and partial autocorrelation function for residuals were checked for no significant correlation.

#### 2.1.1. Univariate Autoregression

This analysis addressed trends in NDVI and the individual climatic factors over the 25-year period. For each 1 km × 1 km pixel, the autoregression model (Proc Autoreg; SAS/ETS, 1999) with stepwise selection for the significant autoregressive error was fitted to the observed values to define the direction and *p*-value for the slope as:

(1) $$Y_{t}=\theta_{0}+\theta_{1}Time+\mu t$$

(2) $$\mu t=\overset{k}{\underset{t=1}{\sum }}\rho_{i}\mu_{t-i}+\varepsilon_{t}$$

(3) $$\varepsilon_{t}\sim IN\left(0,\sigma^{2}\right)$$

where *ϒ* is one of the individual time series variables: monthly NDVI, monthly precipitation, maximum, minimum temperature, and monthly dew point temperature (*n* = 300 months). The fitted autoregression model for the observed variable (*ϒ*_*t*_) is the structural part, which is the same as that of an ordinary least square regression model (OLS; *θ*_0_ + *θ*_1_*Time*)*,* and the autoregressive error (*u*_*t*_). Coefficients *θ*_0_, and *θ*_1_ are the intercept and the slope with time, respectively. The time series error term, *u*_*t*_, may be autocorrelated. The autoregressive error model (Equation (2)) will account for such autocorrelation where the term $\sum_{i}^{k}\rho_{i}\mu_{t-i}$ is the summation of the significant autoregressive parameter (*ρ*) times lagged error(s), and *k* is the order of significant lags in the model. The error term, *ε*_t_, from the autoregressive error model is normally and independently distributed with mean of zero and variance *σ*^2^ (Equation (3)). The slope (*θ*_1_) quantifies the rate and direction of change for each variable over 25 years. We used a significance level of *p* < 0.05 to test whether the slope differed from zero.

#### 2.2.2. Multivariate Autoregression

The multivariate model for each pixel was:

(4) $$NDVI_{t}=\beta_{0}+\beta_{1}P_{t}+\beta_{2}P_{t-1}+\beta_{3}T{\text{min}}_{t}+\beta_{4}T{\text{max}}_{t}+\beta_{5}DP_{t}+\beta_{6}Time_{t}+\mu_{t}$$

(5) $$\mu_{t}=\overset{k}{\underset{t=1}{\sum }}\rho_{i}\mu_{t-i}+\varepsilon_{t}$$

(6) $$\varepsilon_{t}\sim IN\left(0,\sigma^{2}\right)$$

where *P*_*t*_ is precipitation at month *t*, *P*_t−1_ is precipitation for previous month (i.e., one-month lag precipitation), *Tmin* is minimum temperature, *Tmax* is maximum temperature, *DP* is dew point temperature, and *ε* is the error term. The right side of Equation (4) includes the autoregression error (*μ*_*t*_) and the structure term (the remainder of the model), and by summing both these terms yields the predicted value. The estimates (*β*_*i*_*’s*) for each factor quantify the magnitude and direction of the relationship between NDVI and each factor over the 25-year period. The coefficient of Time (*β*_6_) is the temporal trend of NDVI after accounting for climatic factors. We chose blocks of pixels that had either significant increase or decrease (*p* < 0.05) in greenness to aid interpretation of our results by drawing on available literature, consultation with local experts, and Google Earth™.

We considered that potential collinearity among climatic factors may confound the results. However, our primary interest is the significance of the coefficient for the time variable (i.e., NDVI trend). If time is not correlated with any of the climatic factors, the time coefficient variance will not be affected by the collinearity among the climatic factors [61]. From a random sample of 2500 pixels, the correlation between time and each climatic factor was low (lrl ≤ 0.32, r^2^ ≤ 0.10). Consequently, we kept all predictors in Equation (4). In all analyses, we used the 8 bites NDVI values., Only for the temporal NDVI figures, transformed NDVI values (NDVI/255) were displayed.

#### 2.2.3. Comparison of Univariate and Multivariate Models

Because climatic factors were included in the multivariate analysis but omitted in the univariate analysis, a comparison of the results of the univariate and multivariate analyses for each pixel was used to determine if a significant NDVI trend was associated with either indirect factors (climatic factors, and hence, climate change) or direct factors (e.g., fire, agriculture, land cover change). We evaluated four possible outcomes for the significance of NDVI trend in the two analyses. A significant NDVI trend is indicated by a significant time coefficient, i.e., *θ*_1_ in the univariate analysis (Equation (1)) and β_6_ in the multivariate analysis (Equation (4)). The four possible outcomes were:

- NDVI trend was significant in both the univariate and multivariate analyses. NDVI significance apparently resulted from direct factors such as wildfire or agriculture, because the trend was significant regardless of whether climatic factors were included in the analysis.
- NDVI trend was significant in the univariate analysis, but not in the multivariate analysis. This is consistent with a change in the climatic factors as the cause of the significant NDVI trend.
- NDVI trend was not significant in the univariate analysis, but was significant in the multivariate analysis. A significant trend in the multivariate analysis would suggest that the change is presumably due to direct factors. However, the trend in the univariate analysis might not be significant because the variation in NDVI associated indirectly with variation in climatic factors was masked by the influence of direct factors on NDVI.
- NDVI trend was not significant in either the univariate or the multivariate analyses. This would suggest that there is no evidence for a temporal trend in NDVI.

## 3. Results of Univariate and Multivariate Models

### 3.1. Univariate Autoregression Results

**Figure 1.**
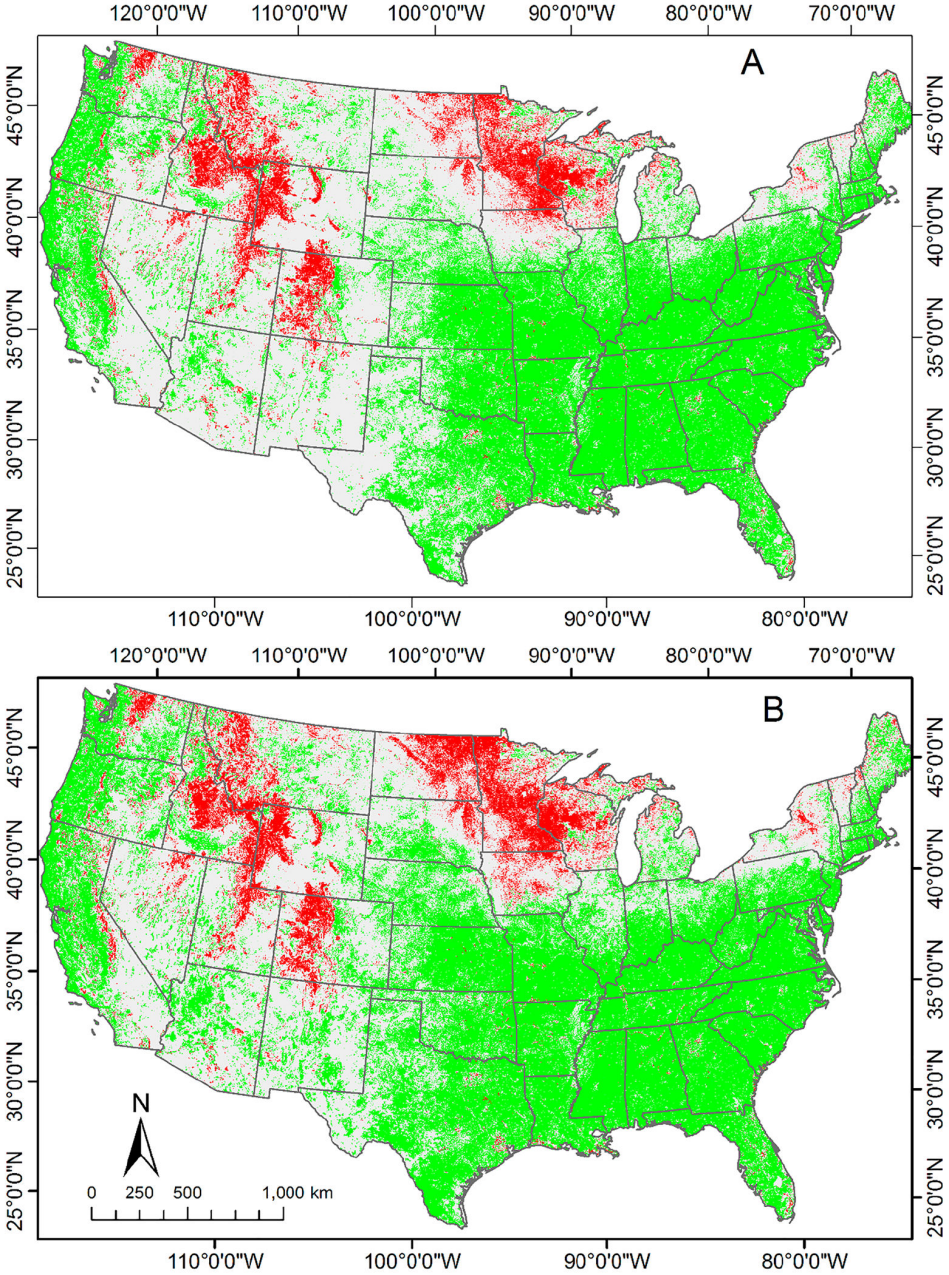
Pixels (1 km × 1 km) with significant temporal trend for the monthly NDVI from 1989 through 2013 in contiguous 48 U.S. states, determined by: (**A**) significant changes in greenness (univariate autoregression; Equation (1)); and (**B**) multivariate autoregression (Equation (4)). Sample size is 300 for each pixel. Green indicates significant *(p* < 0.05) increase in greenness (i.e., NDVI); red indicates significant decrease in greenness.

NDVI changed significantly for approximately one-half of the nation over the 25-year period (1989–2013) in the univariate analyses (Figure 1A; Table 2). The direction of the trend was predominantly positive, 85% of the total significant change was an increase in NDVI (Table 2). This dominant increase in NDVI is occurring across diverse land cover, possible disturbance, practices, and others that we do not have enough information to verify the cause on that scale. Areas with significant greenness increase were concentrated on the pacific coast and southeastern states of the U.S. Areas of significant decrease in greenness were concentrated in the northern and central Rocky Mountains, and west of the Great Lakes.

**Figure 2.**
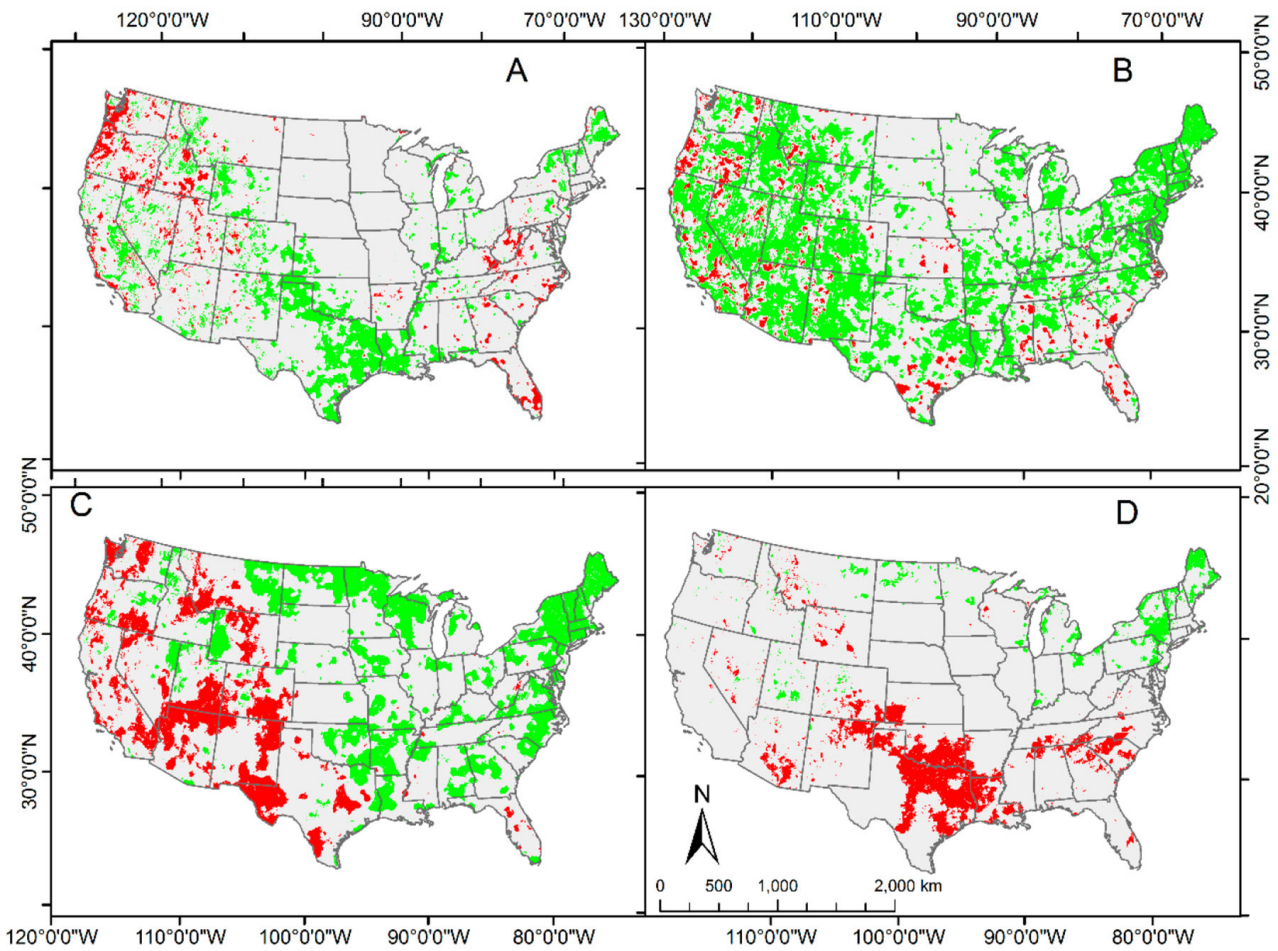
Pixels (1 km × 1 km) with significant temporal trend for monthly climatic factors from 1989 to 2013 in contiguous 48 U.S. states determined using univariate autoregression (Equation (1); *n* = 300 for each pixel). Green indicates significant increase; red indicates significant decrease. Factors are: (**A**) monthly maximum temperature; (**B**) monthly minimum temperature; (**C**) monthly dew point temperature; and (**D**) monthly precipitation.

The proportion of the continental United States with significant changes in monthly climatic parameters ranged from ~12% (precipitation) to ~40% (minimum temperature) (Table 2). Geographic patterns of change differed among the three temperature variables (Figure 2A–C). Significant increases in maximum temperature were concentrated in Texas, Louisiana, and New England (Figure 2A), whereas significant increases in minimum temperature were widespread throughout the continental United States (Figure 2B). Significant decreases in both maximum and minimum temperature were scattered as small clusters throughout the continental United States. Significant increases and decreases in dew point temperature exhibited a geographic dichotomy with increases in the eastern two-thirds of the continental United States and decreases concentrated in the west (Figure 2C). Significant increases in precipitation were concentrated in the northeast and significant decreases were concentrated along the Red River separating Oklahoma and Texas (Figure 2D).

There was not a strong; visual correspondence between locations with significant changes in NDVI and significant changes in the climatic factors. For example, the widespread significant increases in NDVI in the southeastern quadrant of the continental U.S. (Missouri to Florida) (Figure 1A) is accompanied by a much patchier pattern of significant change (increase or decrease) among the climatic factors (Figure 2).

### 3.2. Association between the NDVI and Climatic Factors in Multivariate Analyses

**Figure 3.**
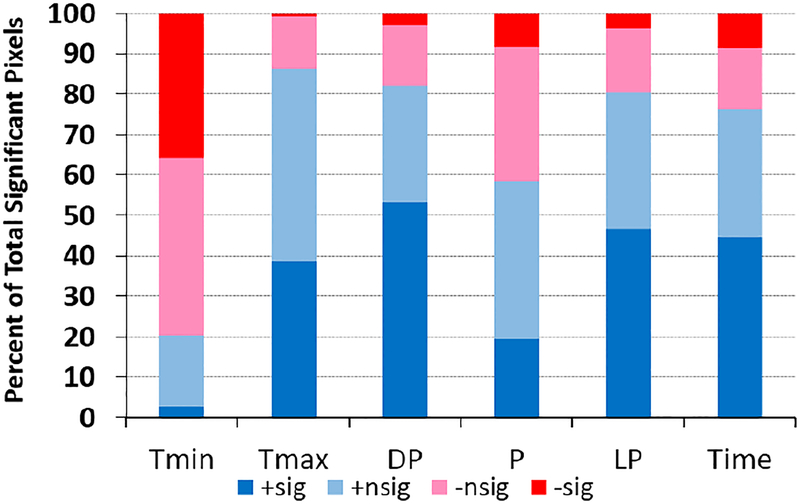
Summary of relationship direction and significance level between the NDVI and climatic factors and time in multivariate autoregression analyses (Equation (4)) for ~7,660,636 1 km × 1 km pixels in USA. Factors are average monthly minimum temperature (Tmin), maximum temperature (Tmax), dew point temperature; (DP), precipitation (P), previous month’s precipitation (Lag[Prcp]), and time. Data are summarized tor pixels with significantly (*p* < 0.05) positive association between the NDVI and the subject factor (+sig), not significant positive association (+nsig), not significant negative (−nsig) association, and significant negative (−sig) association.

NDVI was significantly related to one or more climatic factors in the multivariate autoregression for much of the contiguous 48 states (Figure 3). NDVI was predominantly positively associated with four of the five climatic factors. Particularly frequent positive and significant associations were for dew point temperature (53% of pixels) and 1-month lag precipitation (47% of pixels), with <4% of pixels showing significant negative associations for these two factors. Monthly maximum temperature and precipitation also showed predominantly positive associations with the NDVI, with 39% and 19% of pixels showing significant positive associations, respectively. In contrast to these positive associations, NDVI was negatively associated with minimum temperature (36% of the pixels). The spatial distribution of these significant positive and negative associations between NDVI and the climatic factors are clustered in different patterns across the nation. Precipitation influenced the NDVI proportionally in the central part of the nation arid it doubled with the past month (i.e., one-month Lag precipitation, *P*_t−1_ in Equation (4)) of precipitation.

### 3.3. NDVI Trend in Multivariate Analyses

When climatic factors were included in the multivariate autoregression model, the extent of area with significant NDVI change over the 25-year period increased. NDVI changed significantly for 53% of the pixels in the multivariate autoregression (Table 3; Figure 1B), whereas it changed significantly in only 48% of the pixels in the univariate autoregression (Table 2; Figure 1A). The predominant direction of change in the multivariate autoregression was an increase in NDVI (Figure 1B).

**Figure 4.**
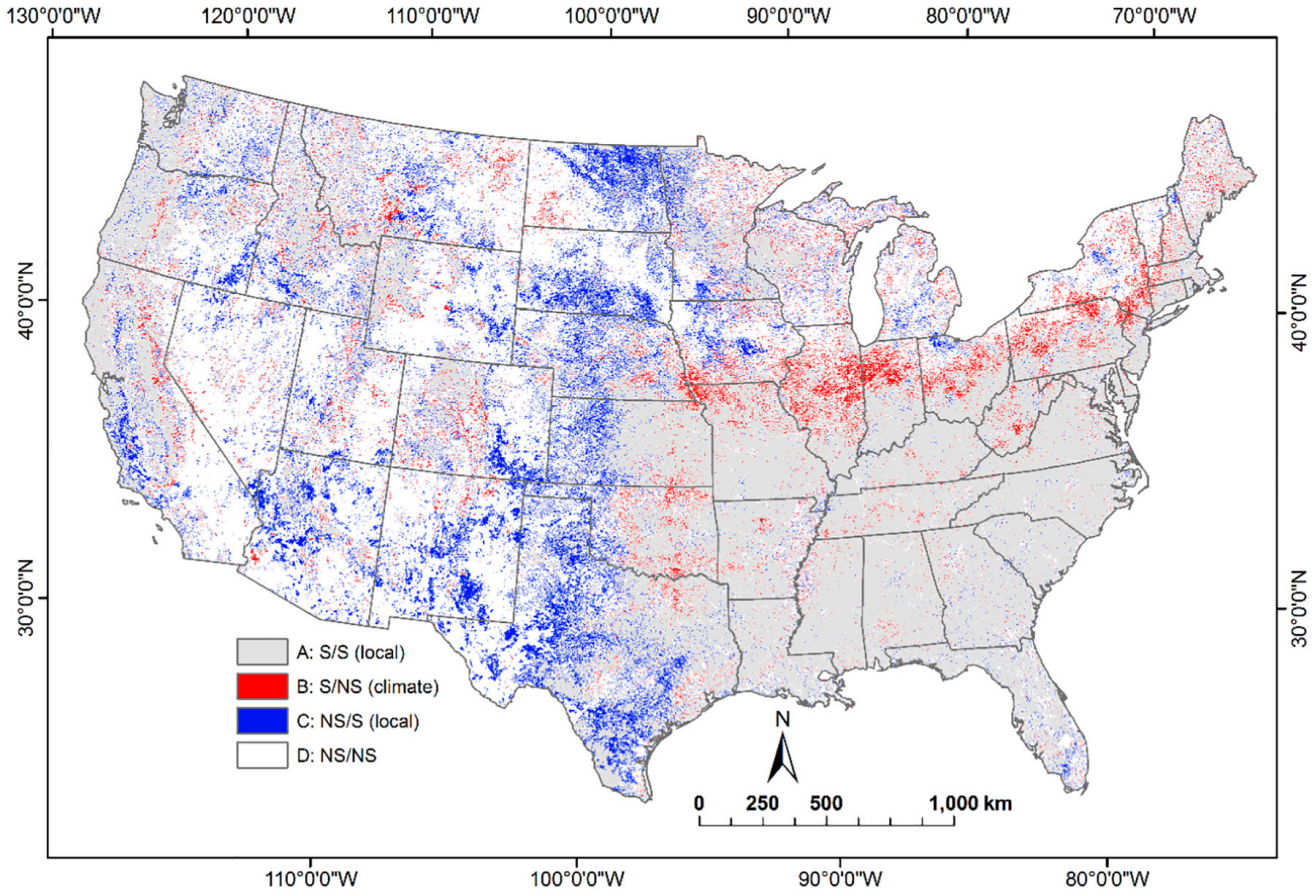
Comparison of the NDVI trend significance between the univariate and multivariate autoregression for each pixel. Outcomes A (significant trend in univariate model/significant trend in multivariate model) and *C* (not significant trend in univariate model/significant trend in multivariate model) denote changes due to direct factors, Outcome B (significant trend in univariate model/not significant trend in multivariate model) denotes changes due to climatic factors, and Outcome D (not significant trend in univariate model/mot significant trend in multivariate model) denotes no change in greenness.

Comparison of NDVI trends between the univariate and multivariate analyses suggests that direct factors were the predominant cause of NDVI change rather than the selected climatic factors (Table 3). The spatial distribution of the four outcomes linking changes in NDVI to that of climatic or direct factor is presented in Figure 4. Outcome A was the most frequent (45%), i.e., there was a significant trend in the NDVI in both analyses and, in this case, all pixels had a trend direction that was the same for both the univariate and multivariate analyses. NDVI significance apparently resulted from factors other than climate, such as direct factors like wildfire, because the trend was significant regardless of whether climatic factors were included in the analysis. A total of 53% (Outcomes A (45%) and C (8%)) of all the pixels were significant for NDVI trend in the multivariate analysis, reflects the influence of the direct factors on NDVI rather than the selected climatic factors. Nearly 4% of the pixels showed climatic factors as the cause of the significant NDVI trend (Outcome B (the trend was significant in the univariate analysis, but not in the multivariate analysis due to inclusion of climatic factors in the multivariate model)), and these pixels tended to cover diverse forested and agricultural land cover (Figure 4). For example, areas with significant increases in NDVI due to climate (Figure 4) extended from eastern Nebraska through Ohio, which is cropland agriculture, and from Pennsylvania through Maine, which is predominantly forested (Figure S1).

## 4. Discussion

Including climatic factors in the multivariate analysis of the NDVI over time increased the percentage of pixels with a significant NDVI trend from 48% (univariate analyses) to 53% (multivariate analyses). Comparisons univariate and multivariate analyses, significant NDVI trends associated with direct factors could be distinguished from other factors. The comparison revealed that NDVI changes were predominantly related to factors other than the climate. Pixels with a significant NDVI trend in either the univariate or multivariate analyses were predominantly related to direct factors as the likely cause of the NDVI trend. Since only 4% at these pixels were in outcome B. Considering the significant changes, the average slope value for Outcome B was less than that of Outcome A but a little higher than that of Outcome C, and the interquartile range (IQR), and the maximum of the trend values for both Outcomes A and *C* were higher than that of outcome 13 (Table S1).

That only a small fraction of the 48 contiguous states had a significant NDVI trend associated with climate change was unanticipated given that several climatic factors have changed significantly during the 25-year study period, and that NDVI was significantly related to these factors in the multivariate autoregression model for much of the area. This small fraction may be due to our distinction between specific climatic and direct factors in the model comparisons. Localized factors such as pest infestations, disease, drought, flooding or shift in agriculture can be in part attributable to climate change [62], but were not included as explicit factors in the multivariate model.

The spatial distribution of the four outcomes linking changes in NDVI to that of climatic or direct factors is presented in Figure 4. The large spatial extent of NDVI change throughout much of the United States that is not related to the climate suggests there are many mechanisms through which vegetation greenness has changed during the 25 years included in the study. More research and study is needed to better understand the broad patterns in NDVI change found in this study. The specific cause for NDVI change in a given area can only be ascertained with detailed knowledge of land cover change in the area, and unfortunately, such data are not readily available for most areas and we will not attempt to explain all the significant changes in NDVI. However, evaluating several areas with some local information provides some understanding for the patterns of the NDVI change specific to those areas.

NDVI in the multivariate autoregression analyses was significantly related to each of the climatic factors analyzed for large fractions of the 48 contiguous states. Thus, given that much of the variation in the NDVI could be accounted for by variation in climatic factors, it is not surprising that a significant NDVI trend was detected at a greater frequency for the multivariate analyses that included climatic factors in comparison to the univariate analyses that excluded them. NDVI was most frequently and positively associated with monthly dew point temperature (53%) followed by previous monthly precipitation (47%). The higher response to previous monthly precipitation than that of the present month was also observed in New Mexico [8] and it is consistent with previous research [63] that found that green-up of grasses occurs several weeks after precipitation. Some of the relationships between NDVI and precipitation and temperature reported here have been observed in other studies [10,64] related to vegetation cover type and spatial locations. The positive association with maximum temperature seems reasonable because warmer temperatures can increase vegetation growth [63,65]. The positive association between the NDVI and maximum temperature presumably reflects the predominant effect of maximum temperature on the NDVI throughout the year, as warmer than average temperatures during summer months would be expected to reduce the NDVI [66]. A strong negative relationship between NDVI and increasing minimum temperature was also observed by [10,67] and found that this relationship is dependent on spatial location, precipitation and growing season. Vegetation response to climatic factors is constrained by geographical location, soil characteristics (e.g., moisture, nutrients, and ground water level), and other factors [68–70]. For example, forest response to warming temperatures is a function of geographic factors (latitude, altitude, aspect, and soil) at a given location [71–73].

## 5. Evaluation of Selected Areas for Cause of NDVI Change

Below, we explore examples of significant change in the NDVI where sufficient information was available to infer the likely cause for the change. The specific examples include significant NDVI changes related to changes due to flooding, fire, and pest infestation. These examples identify areas of dramatic change and rely on information gained from previous in-depth studies and available aerial imagery over the 25-year period that indicates change. One example is given in which significant NDVI change was detected but was attributable to climatic factors instead of a visible direct factor. These examples are not meant to be exhaustive, nor will they explain all NDVI shifts but illustrate the potential use if this autoregressive technique to identify NDVI change and potential links to climatic and direct factors.

### 5.1. Flooding of Agricultural Areas

**Figure 5.**
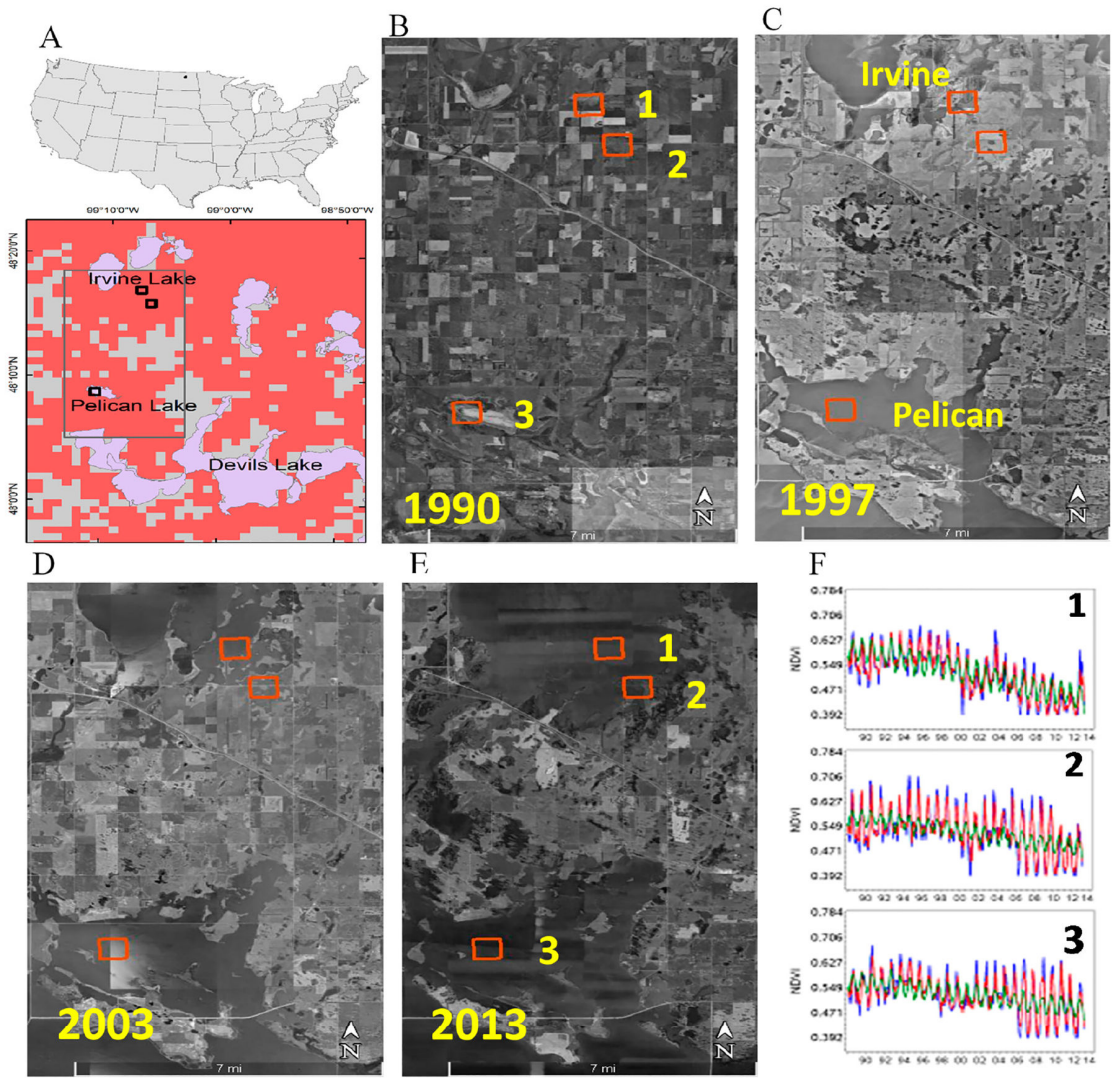
Progression of agriculture and flooding between 1990 and 2013 in Devils Lake area, North Dakota: (**A**) inset map shows the location of the area within the Devils Lake and the selected three numbered 1 km × 1 km pixels; (**B–E**) Google Earth™ images showing the: pre flooding (**A**); and post flooding (**C–E**); and (F) numbered pixels 1–3 in (**A–E**) are used in F1–F3, showing the behavior of NDVI with years. NDVI trend with time (β_6_ < −0.077) is significant (*p* < 0.0001) (lines are described in Figure S2). The coordinates for pixels 1–3 are 48°17′05″N, 99°07′16″W; 48°16′01″N, 99°06′23″W; and 48°10′50″N, 99°10′53″W; respectively.

In the agricultural portions of this region, agricultural practices have generally shifted during the observed time period away from diverse crops and grassland towards corn/soybean rotations [74]. Such shifts in agricultural practices might influence local NDVI responses but the varied responses and drivers make it difficult to identify specific direct causes, especially as these changes are difficult to document or verify with current publicly available imagery. In localized areas where drainage is less prevalent or where drainage is concentrated, more dramatic multi-year changes in flooding on the agriculture landscape can be documented via imagery and might result in decreases in NDVI. NDVI values decrease in areas where agricultural production is stressed or removed due to drought [53,75] or flooding [76–78]. We looked specifically at the agricultural region near Devils Lake, ND where the model indicated direct changes were responsible for changing NDVI (Outcome A) (Figure 5A). The climate of North Dakota and the greater Prairie Pothole Region has strong multi-decadal wet–dry cycles [79]. During the period of 1989–2013, the area shifted from a moderate drought (1988–1992) to a wet period (1993–present) [79,80]. During the drought, lake water levels and lake extent were reduced [81,82] and neighboring lands, and even lake beds, were used for pasture and row crop agriculture. Torrential summer rains in 1993 ushered in the present wet period causing lake levels and lake extents to increase and expand into surrounding fields and grasslands over the; next two decades. By 2011, depressional areas under cultivation and multiple lakes (including Devils Lake, Pelican Lake and Irvine Lake) were all inundated and converged into one large waterbody, increasing the extent of surface water over 400% from 1990 to 2011 [82]. We examined historical aerial imagery around Pelican and Irvine Lake from 1990 to the present for a few representative pixels with significant declines in NDVI (Figure 5). Google Earth™ images show cultivated areas present in 1990 (Figure 5B). In 1997, the pixel at Pelican Lake is inundated while the northern pixels are beginning to wet (Figure 5C). Flooding continues through 2003 (Figure 5D), where all chosen pixels were submerged and show open water by 2013 (Figure 5E). Highest NDVI values were observed around 1993 followed by a gradual decrease of NDVI values (Figure 5F). Vegetation under water stress and turbid open waters typically return a lower NDVI value than those of healthy crops [83].

### 5.2. Fires and Post Fire Greenness Gains

**Figure 6.**
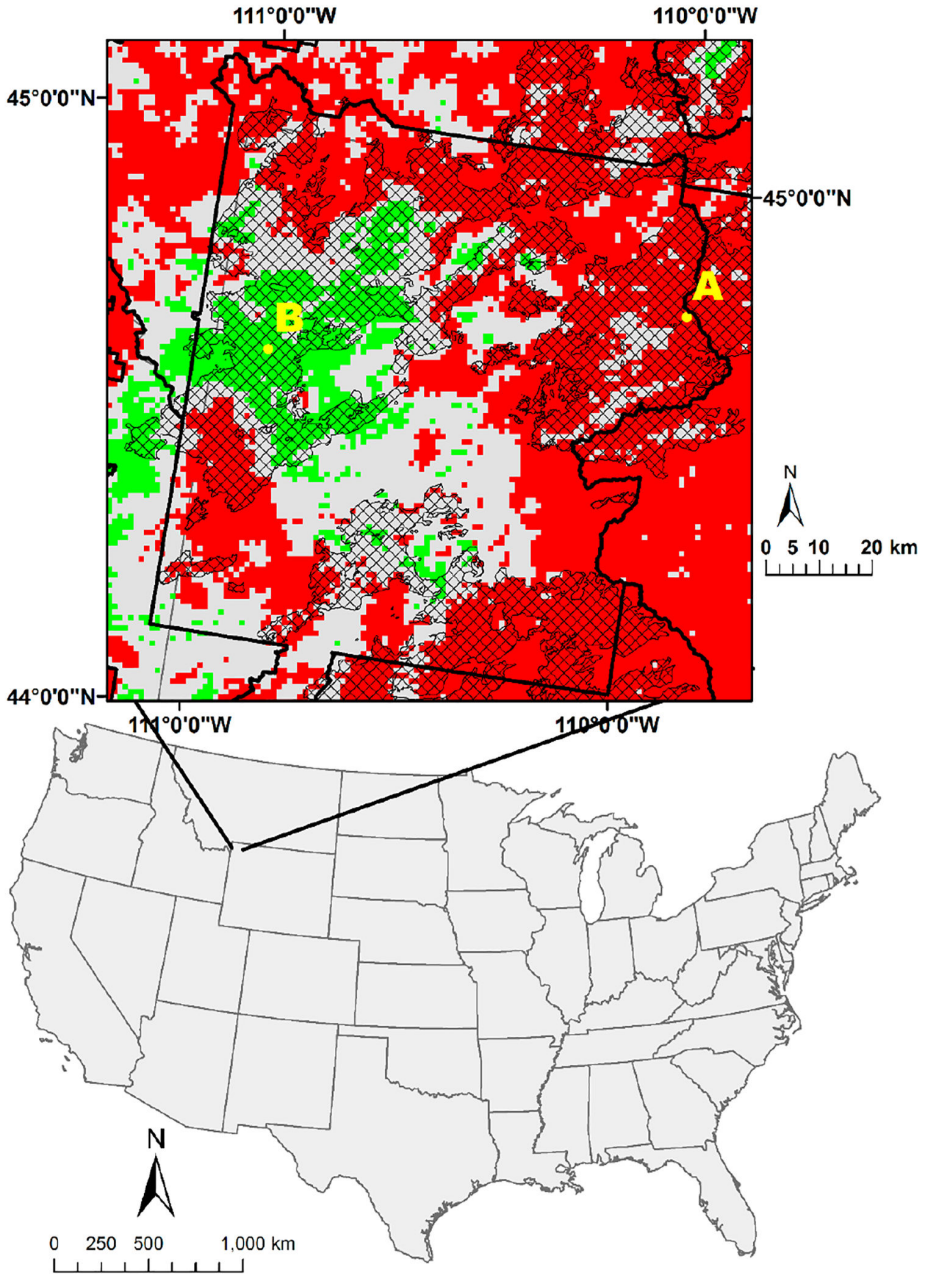
The 1988 Yellowstone fire polygon (black hatching) within Yellowstone National Park (dark black lines). Green and red pixels indicate significant increase and decrease in the NDVI over time (multivariate autoregression), respectively Labeled symbols A and B are the locations of the pixels used in Figures S2 and S3, respectively, showing NDVI behavior over time. (Fire polygon from [87]).

Timing of a fire event and variability in post fire recovery influence the direction of the trend. The effects of fire can produce either a positive or negative NDVI trend depending on when the fire occurred within the time period examined (Figure 6) [8]. In this study, we present an example in Yellowstone National Park where *a* fire occurred in June 1988. The severity of the fire varied spatially [84,85]. Sixty-one percent of the fire affected the forest canopy (crown fire), while 34% occurred as a ground fire [84]. A large fraction of the pixels within and outside Yellowstone National Park experienced a significant decline in the NDVI during the study period except for cluster of significant increase at the western border of the park (Figure 6). All pixels with a significant change (increase and decrease) in NDVI belong to Outcome C (univariate = significant; multivariate = significant), suggesting that change was attributable to direct factors (Figure 4). The pattern of significantly decreasing NDVI is consistent with vegetation loss as a result of the 1988 fire just prior the onset of the NDVI period reported here (1989–2013) followed by a reduced NDVI for many years due to limited vegetation recovery, as represented by the pixel located at A in Figure 6 (see Figure S2). Pixel A is located in higher elevation where the influence of soil moisture and temperature on vegetation is more than on B. Additionally, trees with canopy fires become more susceptible to insect infestation from infested nearby trees [86]. In contrast, vegetation recovery has occurred since the 1988 fire in the block of pixels with significantly increasing NDVI at the western edge of the park boundary, as represented by the pixel located at B in Figure 6 (see Figure S3).

### 5.3. Tree Mortality Due to Insect Infestation

**Figure 7.**
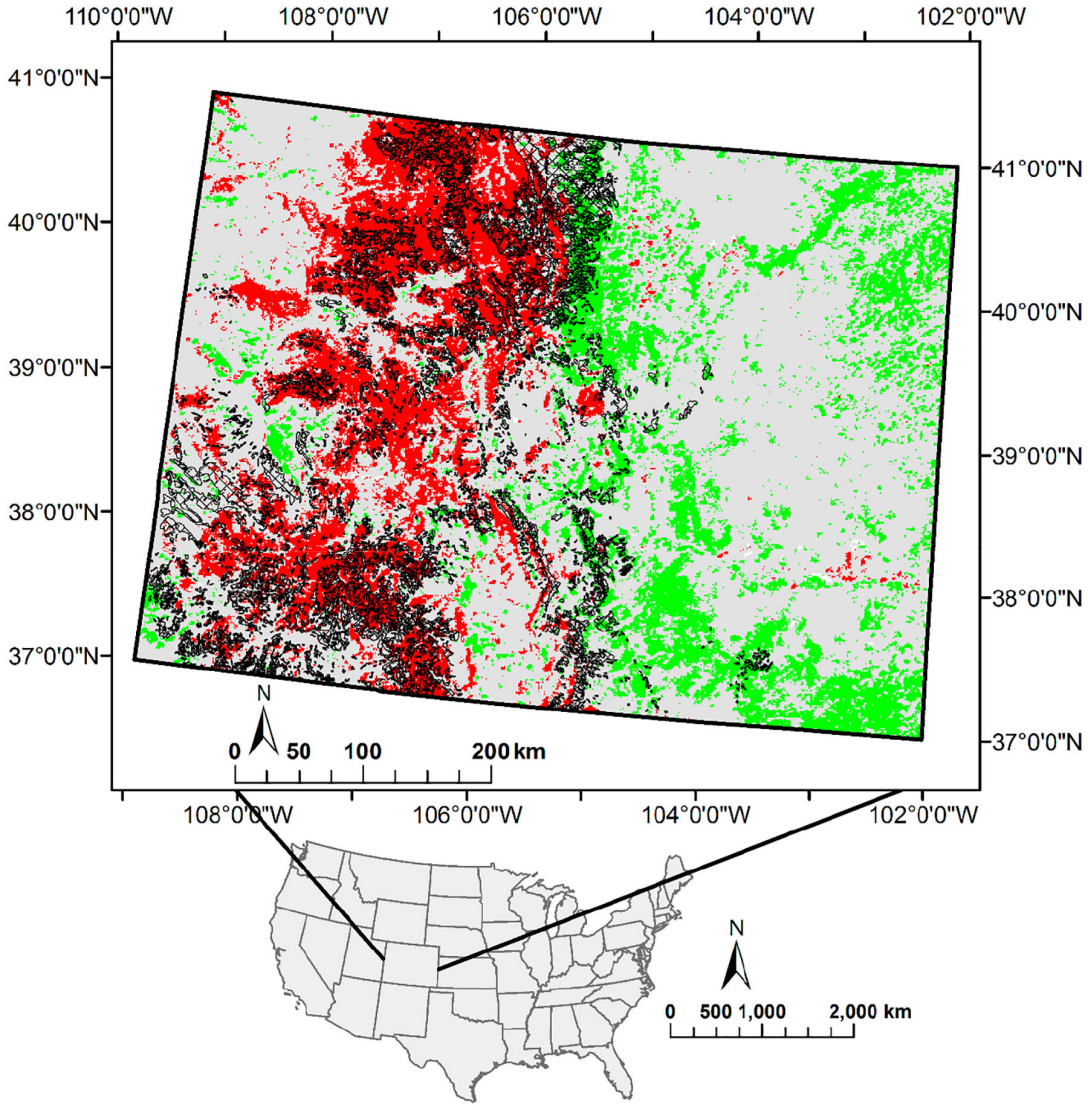
Progress of tree mortality from insect infestation and diseases in Colorado from 1994 to 2013 (black hatching). Green pixels indicate a significant increase in the NDVI over time (multivariate autoregression), and red pixels indicate significant decrease in NDVI over time (multivariate autoregression).

Many forested areas in the USA have suffered substantial tree mortality and defoliation from pest infestations and diseases [18,88–90] which have been particularly severe in Colorado [91]. The occurrence of tree mortality is largely coincident with decreases in NDVI (Figure 7). All pixels with significantly changing NDVI in Figure 7 are attributable to direct: factors though direct factors like insect infestations can be influenced by changes in climate factors. Precipitation for consecutive years was below normal in the infested area [91]. The association of insects with hosts is dependent on climatic factors and varies with the geographical location [91,92] and insect infestation increased due to reduced resistance in drought-stressed trees. In this area, precipitation with no significant change while minimum temperature increased significantly during the study period in the mortality area. An increase of the minimum temperature reduces the likelihood of extreme low temperatures which would set back pest infestations [86]. Dew point temperature also decreased significantly in parts of the infested areas.

### 5.4. Change Attributable to Climate

**Figure 8.**
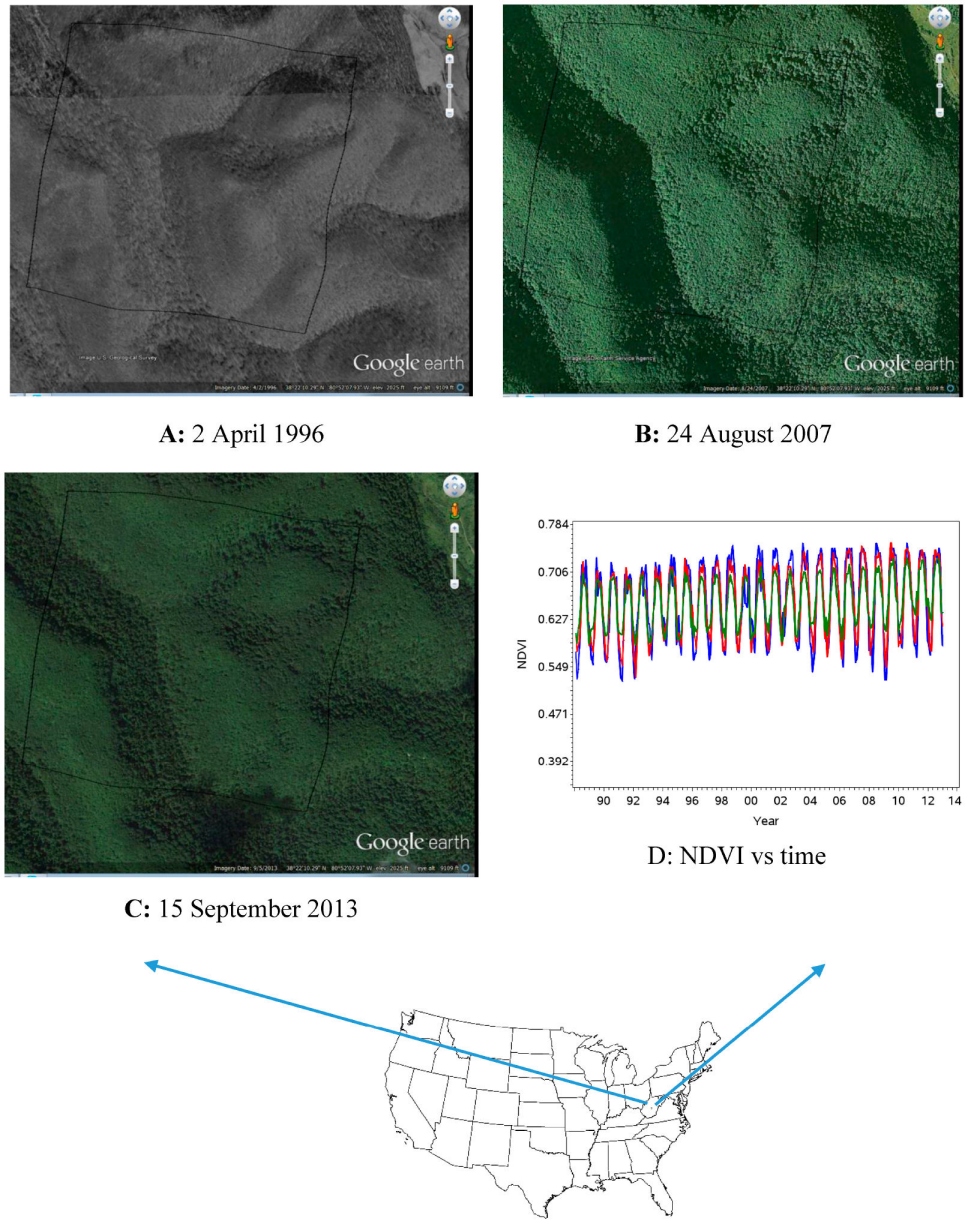
Forested land located in central West Virginia that had a significantly increasing NDVI trend without discernable land cover change (Outcome B). The latitude and longitude of the pixel center is 38°22′11″N and 81°51′56″W. (**A–C**) Google Earth™ images indicating no change in land cover between 1996 and 2013; and (**D**) NDVI with time (NDVI trend increased significantly in univariate (*p* < 0.0001) but it was not significant after including the climatic factor (*p* = 0.0663) (lines are described in Figure S2).

Unlike the examples shown for change attributable to direct factors, for pixels in outcome B, we would not expect to find land cover changes that would be visibly discernible in maps and aerial photography. We provide one example (Figure 8) from a location in central West Virginia for which there was aerial imagery from 1996 to 2013. For this location, there was no apparent change in land cover or vegetation vigor between 1996 and 2013 even though there was a significantly increasing trend in NDVI. The comparison between time coefficients from univariate and multivariate models are presented in Figures S4 and S4. The trend value and its significant p value for the climatic factors and NDVI are given is Table S2. From the multivariate autoregression model, NDVI associated positively and significantly with dew point temperature (coefficient = +2.6152, *p* < 0.0001) and responded negatively and significantly to minimum temperature (coefficient = −1.8981, *p* = 0.0025). Responses of NDVI were not significant with maximum temperature, precipitation and one-month lag precipitation.

### 5.5. Comparison with Previous Studies

It is possible to compare our results with two studies [36,49] listed in Table 1. Comparison with the others is not possible because of locational mismatches or lack of quantification of long-term NDVI trends over broad geographic extents. Using the 4-km GVIx AVHRR data for the period 1982–2007, [49] found significantly decreasing NDVI trends in shrublands and significantly increasing trends in grasslands across the Great Plains and western United States. Using the 8-km GIMMS AVHRR data from 1981 through 2007, [36] found extensive areas of significantly decreasing NDVI along the USA-Mexico border (central Texas to California) that extended north along the California coast to San Francisco, and areas of significantly increasing NDVI in a “sea” of non-significant NDVI changes throughout the semi-arid western United States. These results [36,49] are not fully consistent with the results reported here. For our univariate and multivariate results, we found a distinct concentration of significantly decreasing NDVI in the forested (i.e., higher elevations) portion of the Rocky Mountains and isolated areas of both significantly increasing and decreasing NDVI in the more arid lower elevations of the western United States in which the areas of significantly decreasing NDVI tended to be smaller and more isolated than the areas of significantly increasing NDV I (Figure 3). Although speculative, the differences between our results and those of [36,49] are probably attributable to differences in the resolution of the AVHRR NDVI data sets used, differences in the time period examined, and differences in the statistical methods used to quantify NDVI trends. Our results appear to be finer grained (i.e., patchy) and we suspect that this is attributable to the higher resolution of the AVHRR NDVI data we used. Although relationships between earth surface features and sensors are complex and dynamic, a general rule-of-thumb is that sensor systems do not tend to resolve features smaller than twice the size of the sensor spatial resolution [93,94]. Our AVHRR NDVI data has 16 pixels for every single pixel in the GVIx data and 64 pixels for every single pixel in the GIMMS data, suggesting that the AVHRR data we used were better able to discern spatial patterns at a finer scale than the coarser resolution AVHRR data used previous studies. The time period of our study was substantially different than the other two, starting and ending about 6–8 years later [36,49]. Temporal differences in the time period examined would likely result in differences in the spatial pattern of NDVI trend significance since the factors attributable to NDVI changes are also temporally dynamic. We would expect to find greater similarity between our results and those of [36,49] if they were calibrated to the same period (e.g., 1989–2007) despite differences in data sources and statistical methods. The time period chosen for our study was dictated by the availability of the high resolution AVHRR data we used [9]. It is also intuitive that the choice of statistical methods would influence the outcome. Neither [49] nor [36] used classical autoregressive techniques to account for temporal correlation. Linear regression of NDVI versus precipitation was used to detect trends in [49] and in [36], Mann–Kendall and linear correlation were used to detect inter-annual trends, and harmonic regression, a technique related to autoregression, was used to uncover intra-annual periodicities. We used long-established statistical methods that account for serial correlation in temporal data so that parameter standard errors and hence significance were evaluated correctly

## 6. Conclusions

Remote monitoring of changes in greenness over time can be useful for identifying long term trends resulting from climate change or anthropogenic activities on the ground. The effect of different climatic factors (temperature and precipitation) and anthropogenic factors on NDVI is not homogenous across space and time. The present study shows that NDVI is often significantly related to precipitation and temperature, and that the relationships are not necessarily intuitive. Consequently, climatic factors may confound the ability to detect areas of NDVI change not associated with climatic factors. By including climatic factors in a multivariate analysis of the NDVI over time, the detection of areas with significant NDVI change can be increased. Moreover, a comparison of analyses with and without climatic factors can be used to distinguish between areas of NDVI change associated to climatic factors and areas associated with direct factors. Comments about changes of slope and once the areas affected by direct factors have been identified, they can be evaluated for causes of change, such as fire or fire recovery, change in flooding extent, change in agriculture extent or practices, and insect infestations. A closer look and monitoring changes in trends direction and magnitude in a specific affected area can be segmented according to disturbance time as we presented in this paper. This could support tailored management strategies based on the major causes of direct and/or climate change in the area.

## Supplementary Material

sup

## Figures and Tables

**Table 1.** Recent studies of AVHRR NDVI—climate data relationships.

Reference	Year	Location	Length	AVHRR Data	AVHRR Resolution	Climate Data Resolution	Independent Variables	Analysis
[32]	2009	Africa	1982–1996	GIMMS	8-km	0.5°	P	OLS
[33]	2007	Tibetan Plateau, China	1982–1989	GIMMS	8-km	Station	P	LC
[34]	2011	China	1982–2006	GIMMS	8-km	Station	T,P	OLS
[35]	2009	Brazil	1981–2006	GIMMS	8-km	0.5°	P	OLS
[36]	2012	Drylands, Global	1981–2007	GIMMS-g	8-km	2.5°	T,P,E	MK, OLS
[37]	2005	Sahel, Africa	1982–2003	GIMMS	8-km	2.5°	P	OLS
[38]	2002	Global	1982–1990	PAL	8-km	0.5°	T,P	OLS
[39]	2004	USA	1989–1993	EROS	1-km	Station	T,P,PET, E	DW, spR
[40]	2010	USA and Canada	1981–2001	PAL	8-km	0.25°, 32-km	T,P,LST,E,S	OLS
[41]	2012	Canada	1985–2007	PAL	1-km	Station	T, P	MK, OLS
[42]	2012	China	1982–2003	GIMMS	8-km	Station	T, P	OLS
[43]	2012	Mexico	1982–2007	GIMMS	8-km	Station	P	MK, HR, visual
[44]	2005	Sahel, Africa	1982–1999	PAL	8-km	2.5°	P	visual
[45]	2010	Asia	1982–2006	GIMMS	8-km	2.5°	T, P	EOF, SVD
[46]	2012	China	1982–2012	GIMMS	8-km	0.1°	T, P	OLS
[47]	2009	Brazil	1982–2006	GIMMS	8-km	0.1°	P	visual
[48]	2004	USA	1990–2000	EROS	1-km	Station	SOI, P	visual
[49]	2010	USA	1982–2007	GVIx	4-km	32 km^2^	DSL, P	OLS
[50]	2008	S. America	1981–2000	GIMMS	8-km	-	Water level	visual

Notes: blank entry = not reported; GIMMS = Global Inventory Modeling and Mapping Studies; PAL = Pathfinder AVHRR Land; station = climate data from local weather stations; T = temperature; P = precipitation, PET = potential evapotranspiration; E = solar radiation; S = soil moisture; LST = land surface temperature; SOI = southern oscillation index; DSL = dry season length; OLS = ordinary least squares; LC = linear correlation; MK = Mann–Kendall; DW = Durbin–Watson; spR = spatial regression; visual = qualitative; EOF = empirical orthogonal function; SVD = singular value decomposition.

**Table 2.** Percent of total pixels with significant increasing or decreasing trend in the univariate analyses for the NDVI and for each of the climatic factors. Significance level is *p* < 0.05.

Variable	Increase	Decrease	Total
NDVI	41.11	7.28	48.38
Minimum Temperature	35.41	4.09	39.50
Maximum Temperature	11.94	4.45	16.39
Dew Point	20.08	12.52	32.60
Precipitation	2.88	8.74	11.62

**Table 3.** Frequency of outcomes in comparison of NDVI trends; between univariate and multivariate analyses for ~7,660,636 1 km × 1 km pixels in conterminous states. Outcomes A and C denote changes due to direct factors, Outcome B denotes changes due to climatic factors, and Outcome D denotes no change in greenness.

NDVI Trends in Univariate Analysis	NDVI Trends in Multivariate Analyses		
	Significant	Not S ignificant	Total
Significant	Outcome A 44.83	Outcome B 3.56	48.38
Not Significant	Outcome C 8.16	Outcome D 43.45	51.62
Total	52 at	47.01	100.00
