# Supplementary material for: Changes in Landscape Greenness and Climatic Factors over 25 Years (1989–2013) in the USA
Source: Remote Sens (Basel). Author manuscript; Available in PMC 2018 Nov 28. (PMC6261326; doi:10.3390/rs9030295)
Supplement: sup [file NIHMS983410-supplement-sup.pdf]

remote sensing

MDPI

Article

Changes in Landscape Greenness and Climatic Factors over 25 Years (1989–2013) in the USA

Maliha S. Nash [1] *, James Wickham [2], Jay Christensen [1], and Timothy Wade [3]

We used the AVHRR 1 km^2 local area coverage (LAC) data that are available for the continental United States for our analysis [1]. AVHRR data processing has been ongoing since 1989 [2], and it constitutes the only consistently processed 1 km^2 AVHRR data for the globe. Processing of these data include radiometric correction that results from sensor degradation, adjustments for atmospheric effects, and geometric registration accuracy. The 1 km^2 AVHRR LAC data are produced from NOAA-11, -14, -16, and -17 satellites. Radiometric corrections are based on sources specific to each satellite (see Table 1 in [1]). Adjustments for atmospheric effects include corrections for ozone, water vapor absorption, and Rayleigh scattering. Water vapor absorption can reduce near-infrared band reflectance by up to 30%, and Rayleigh scattering and ozone absorption can increase reflectance in the red band by up to 2% [1]. Geometric registration is accomplished using image-to-image registration rather than image to map registration because the former improved geometric accuracy. All observations used in the geometric registration must have a root mean square error of less than 1 pixel. On average, 10 satellite passes per week are used to develop the NDVI maximum value composite (MVC) data. The maximum value composite data methods were developed by [3].

The climatic factors selected represent precipitation and temperature because these two factors are generally strongly associated with greenness [4,5]. We also included dew point temperature, which is the temperature at which atmospheric moisture condenses, because it reflects both temperature and moisture conditions, and it was shown to be positively related to NDVI in a study of agricultural and residential areas in Phoenix, Arizona [6]. We used five climatic factors, four of which were original and one of which was derived. The four original variables were monthly averages of precipitation, maximum temperature, minimum temperature, and dew point temperature. The fifth variable was one-month lagged precipitation, derived from monthly precipitation. The four original climate factors were obtained from Parameter-elevation Regressions on Independent Slopes Model (PRISM; http://www.prism.oregonstate.edu/products/matrix.phtml, accessed March 2012) using observed climatic data from > 10,000 stations [7]. The 4km × 4km grid cells of the interpolated PRISM climatic variables were gridded into 1km x 1km grid cells using the inverse distance weighted method in ARC-GIS 9.3.1 (ESRI, Redlands, California) to match the NDVI resolution. Downscaling of PRISM climate data from 4km × 4km to 1km × 1km was applied recently by [8]. In this study we are using one-month lagged precipitation due to its stronger relation with NDVI [9, 10]. For the model, we used the monthly NDVI, which is the maximum NDVI value of the biweekly NDVI derived by applying MapAlgebra in ARC-GIS 9.3.1. In all analyses, we used the 8 bites NDVI values, only for temporal NDVI figures, transformed NDVI values (NDVI/255) were displayed.

1. Supplementary Tables

Table S1. Statistical summary for the trend values per outcome. Outcomes A and C denote changes due to direct factors, Outcome B denotes changes due to climatic factors, and Outcome D denotes no change in greenness. S denotes significant, u denotes univariate, m denotes multivariate, n denotes not. Percent (%) is the proportion of outcomes as described in Table 3.

Outcome	%	Min	P25	Mean	Median	P75	IQR	Max
A(Su/Sm)	44.83	-0.5690	0.0193	0.0180	0.0275	0.0357	0.0164	0.1764
B(Su/nSm)	3.56	-0.0814	0.0107	0.0091	0.0147	0.0182	0.0075	0.0802
C(nSu/Sm)	8.16	-0.1030	0.0001	0.0062	0.0119	0.0163	0.0163	0.0857
D(nSu/nSm)	43.45	-0.0818	-0.0025	0.0025	0.0036	0.0083	0.0108	0.0846

Table S2. Trend value, trend direction and the significant p-value for the climatic factors from the univariate autoregression (Equation (1)). The last two rows present NDVI trend, direction and *p*-value from univariate, and multivariate models.

Variable	Trend Value	*p*-value
P	−0.0086	0.88
Tmin	+0.0020	0.21
Tmax	−0.0006	0.63
Dp	+0.0020	0.15
NDVI(univariate)	+0.0276	0.0001
NDVI(multivariate)	+0.0287	0.066

2. Supplementary Figures

The land cover (Figure S1) is dominated in close proportion by forest (26%) and shrubland (23%). While cultivated crop (16%) and grass (15%) are close in their covers, wetland and emergent wetland cover 5% [11] (Fry et al., 2011). Labeled symbols A and B in Figure 6 are the locations of the pixels within the Yellowstone used in Figs. S2 and S3, respectively, showing NDVI behavior over time. The severity of the fire on greenness varied spatially. Following changes in Greenness post fire, we observe that pattern of significantly decreasing NDVI (β6 < -0.077, $p < 0.0001$) is consistent with vegetation loss as a result of the 1988 fire just prior the onset of the NDVI period reported here (1989–2013) (Figure S2). In contrast, vegetation recovery has occurred since the 1988 fire in the block of pixels with significantly increasing NDVI at the western edge of the park boundary, as represented by the pixel located at B in Figure 6 (Figure S3, β6 = 0.0623, $p = 0.0002$).

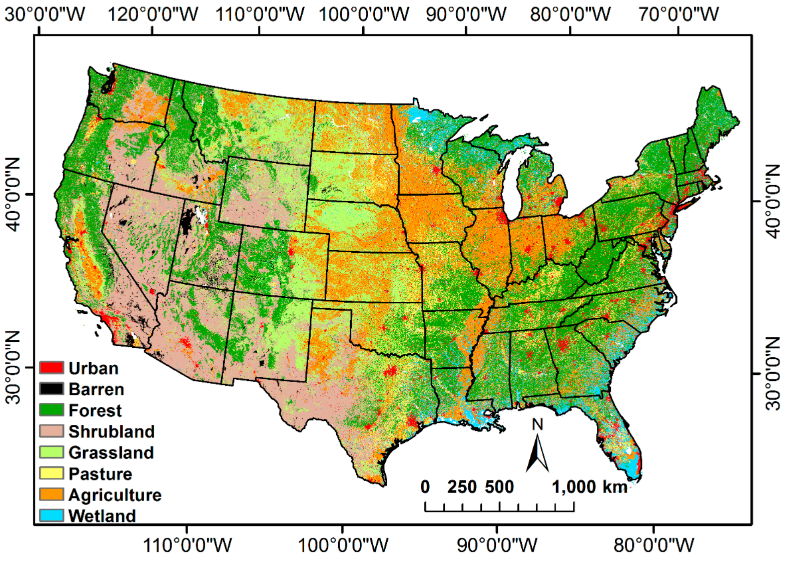

Figure S1. Land cover for the conterminous US. Data from National Land Cover Database (Fry et al., 2011).

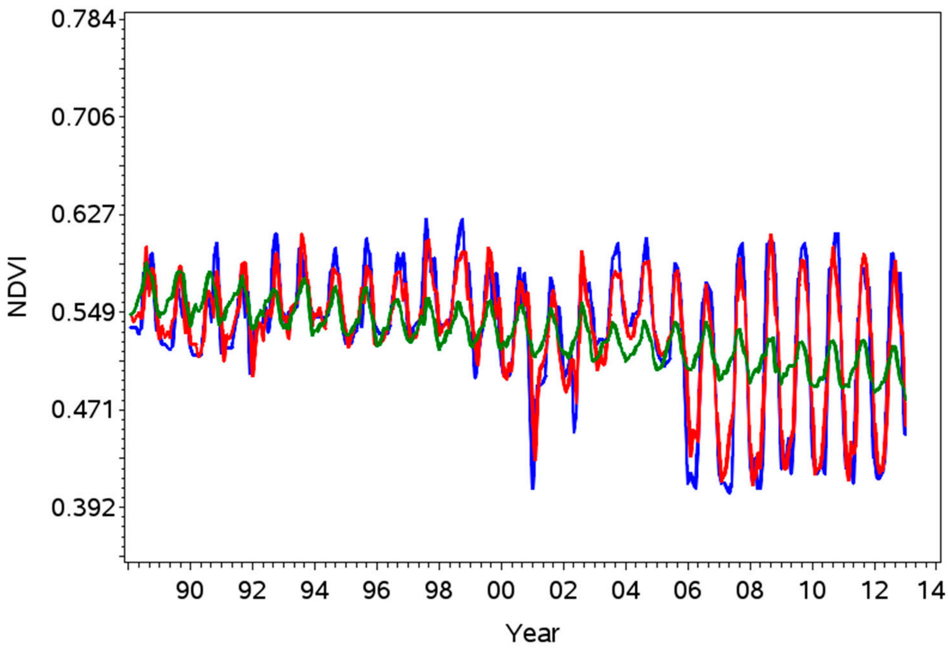

Figure S2. Observed and predicted values for NDVI for one pixel in the Yellowstone Fire (pixel A in Figure 6). The blue line is the observed values (n=300). The red line is the predicted values from the multivariate autoregression model and the green line represent the structural part of the multivariate autoregression model (see explanation for Equation (4)). NDVI trend with time (β_6 = -0.0604) is significant ($p < 0.0001$).

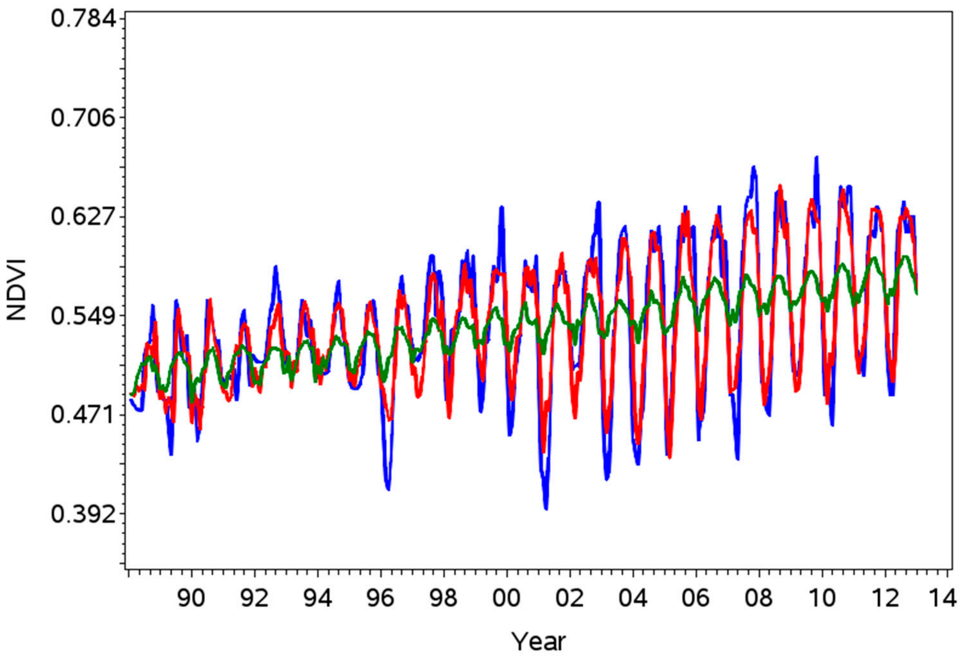

Figure S3. Observed and predicted values for NDVI for one pixel in the Yellowstone Fire (pixel B in Figure 6). The blue line is the observed values (n=300). The red line is the predicted values from the multivariate autoregression model and the green line represent the structural part of the multivariate autoregression model (see explanation for Equation (4)). NDVI trend with time (β_6 = 0.0623) is significant ($p = 0.0002$).

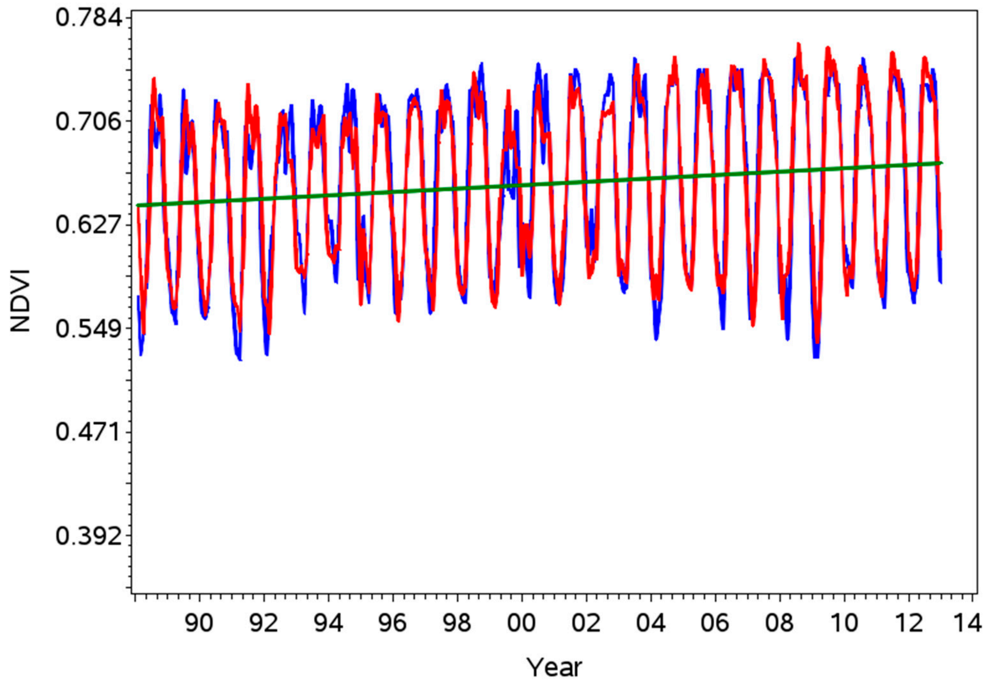

Figure S4. Observed and predicted values for NDVI for one pixel within outcome B (see Figure 8). The blue line is the observed values (n=300). The red line is the predicted values from the univariate autoregression model and the green line represent the coefficient of time of the univariate autoregression model (see explanation for Equation (1)). NDVI trend with time (θ_1 = 0.0276) is significant ($p < 0.0001$).

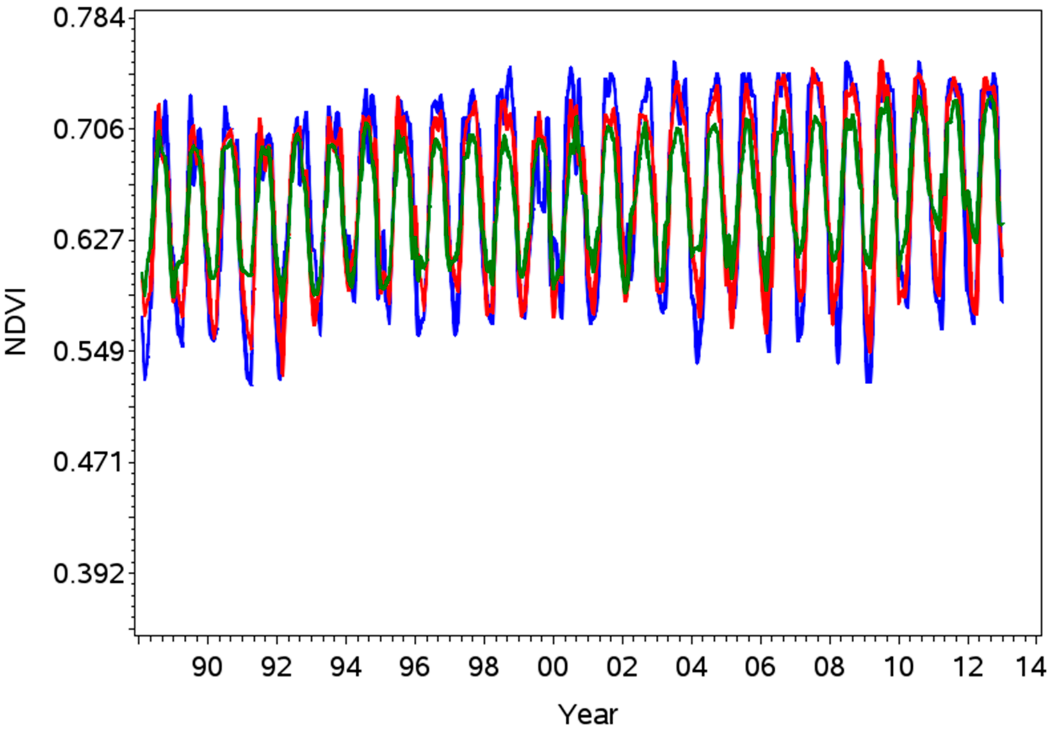

Figure S5. Observed and predicted values for NDVI for one pixel within outcome B (see Figure 8). The blue line is the observed values (n=300). The red line is the predicted values from the multivariate autoregression model and the green line represent the coefficient of time of the multivariate autoregression model (see explanation for Equation (4)). NDVI trend with time (β_6 = 0.0281) is not significant ($p = 0.0663$).

References

1. Eidenshink, J. A 16-year time series of 1 km AVHRR satellite Data of the conterminous United States and Alaska. *Photogramm. Eng. Remote Sens*. **2006**, *72*, 1027–1035.
2. Eidenshink, J.C.; Faundeen, L. 1-KM AVHRR global land dataset: first stages of implementation. *Int. J Remote Sens.* **1994**, *35*, 3443–3462.
3. Holben, B.N. Characterization of maximum-value composite images from temporal AVHRR data. *Int. J Remote Sens.* **1986**, *7*, 1417−1434.
4. Wang, J.; Rich, P.M.; Price, K.P. Temporal responses of NDVI to precipitation and temperature in the central Great Plains, USA. *Int. J Remote Sens.* **2003**, *24*, 2345–2364.
5. Twumasi, Y.A.; Coleman, T.L.; Manu, A.; Merem, E.C.; Osei, A. Relationship between climate parameters and forest vegetation at and near Digya National Park, Ghana. *Br. J. Environ. Climate Chang*. **2011**, *1*, 201−215.
6. Stabler, L.B.; Martin, C.A.; Brazel, A.J. Microclimates in a desert city were related to land and vegetation index. *Urban For. Urban Green*. **2005**, *3*, 137–147.
7. Daly, C.; Halbleib, M.; Smith, J.; Gidsin, W.P.; Doggett, M.K.; Taylor, G.H.; Curtis, J.; Pasteris, P.P. Physiological sensitive mapping of climatological, temperatures and precipitation across the conterminous United States. *Int. J. Climatol*. **2008**, *28*, 2031-2065.
8. Thorne, J.; Boynton, R.; Flint, L.; Flint, A.; Le, T. Development and Application of Downscaled Hydroclimatic Predictor Variables for Use in Climate Vulnerability and assessment Studies. California Energy Commission. Publication number: CEC-500-2012-010, 2012.
9. Nash, M.S.; Bradford, D.F.; Wickham, J.D.; Wade, T.G. Detecting Change in Landscape Greenness over Large Areas: An Example for New Mexico, USA. *Remote Sens. Environ*. **2014**, *150*, 152-162.
10. Notaro, M.; Liu, Z.; Gallimore, R.G.; Williams, J.W.; Gutzler, D.S.; Collins, S. Complex seasonal cycle of ecohydrology in the southwest United States. *J. Geophys. Res*. **2010**, *115*, pp. 1–20.
11. Fry, J.; Xian, G.; Jin, S.; Dewitz, J.; Homer, C.; Yang, L.; Barnes, C.; Herold, N.; Wickham, J. Completion of the 2006 National Land Cover Database for the Conterminous United States. *Photogramm. Eng. Remote Sens*. **2011**, *77*, 858−864.
